# Supplementary figures and images for: Hypergraph models of biological networks to identify genes critical to pathogenic viral response
Source: BMC Bioinformatics. 2021 May 29;22:287. doi: 10.1186/s12859-021-04197-2 (PMC8164482; doi:10.1186/s12859-021-04197-2)

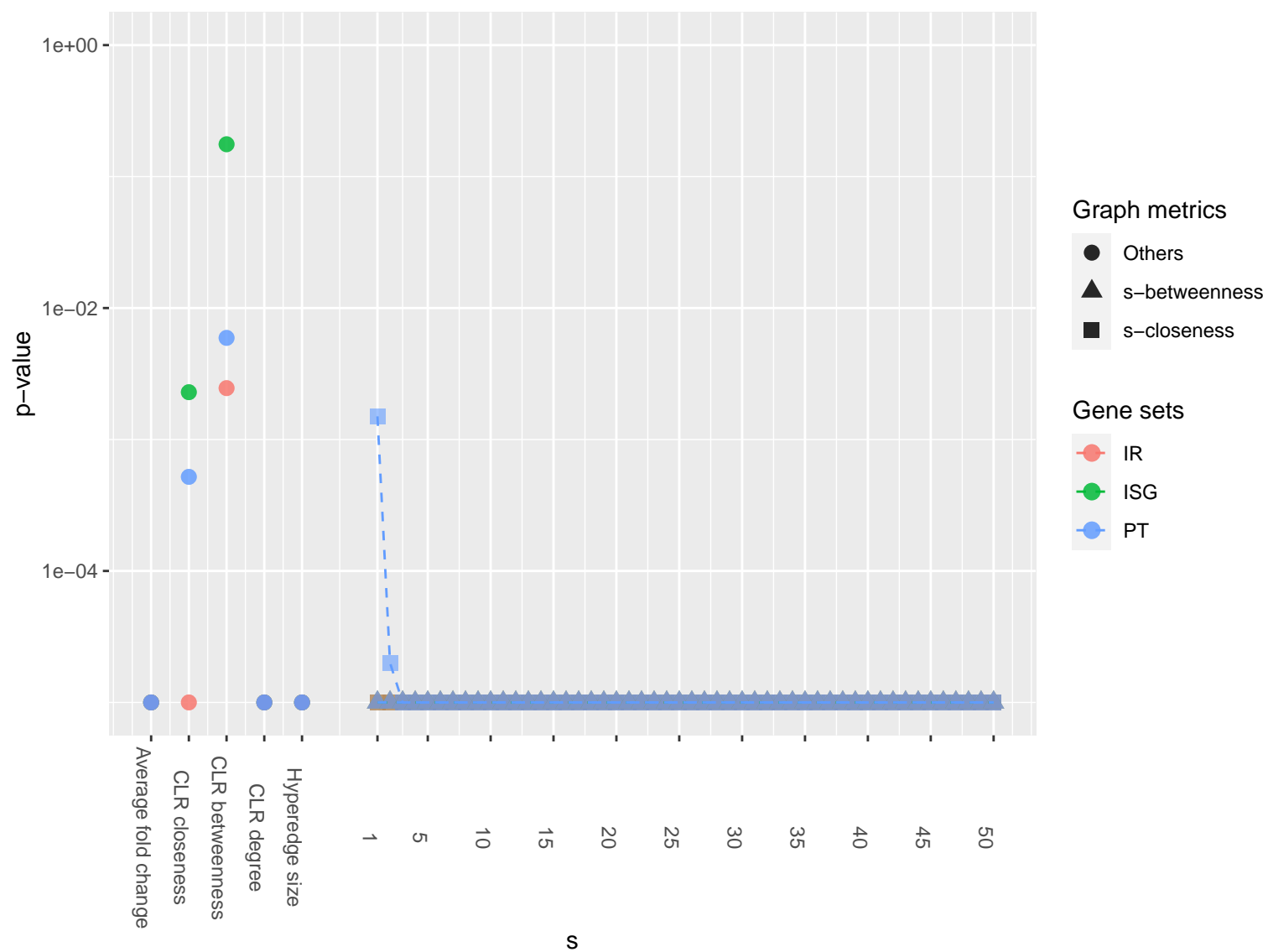

Supplement: Supplementary file 2 — Additional file 2. These figure is analogous to Figure 4 for additional z-score threshold z. [file 12859_2021_4197_MOESM2_ESM.pdf]

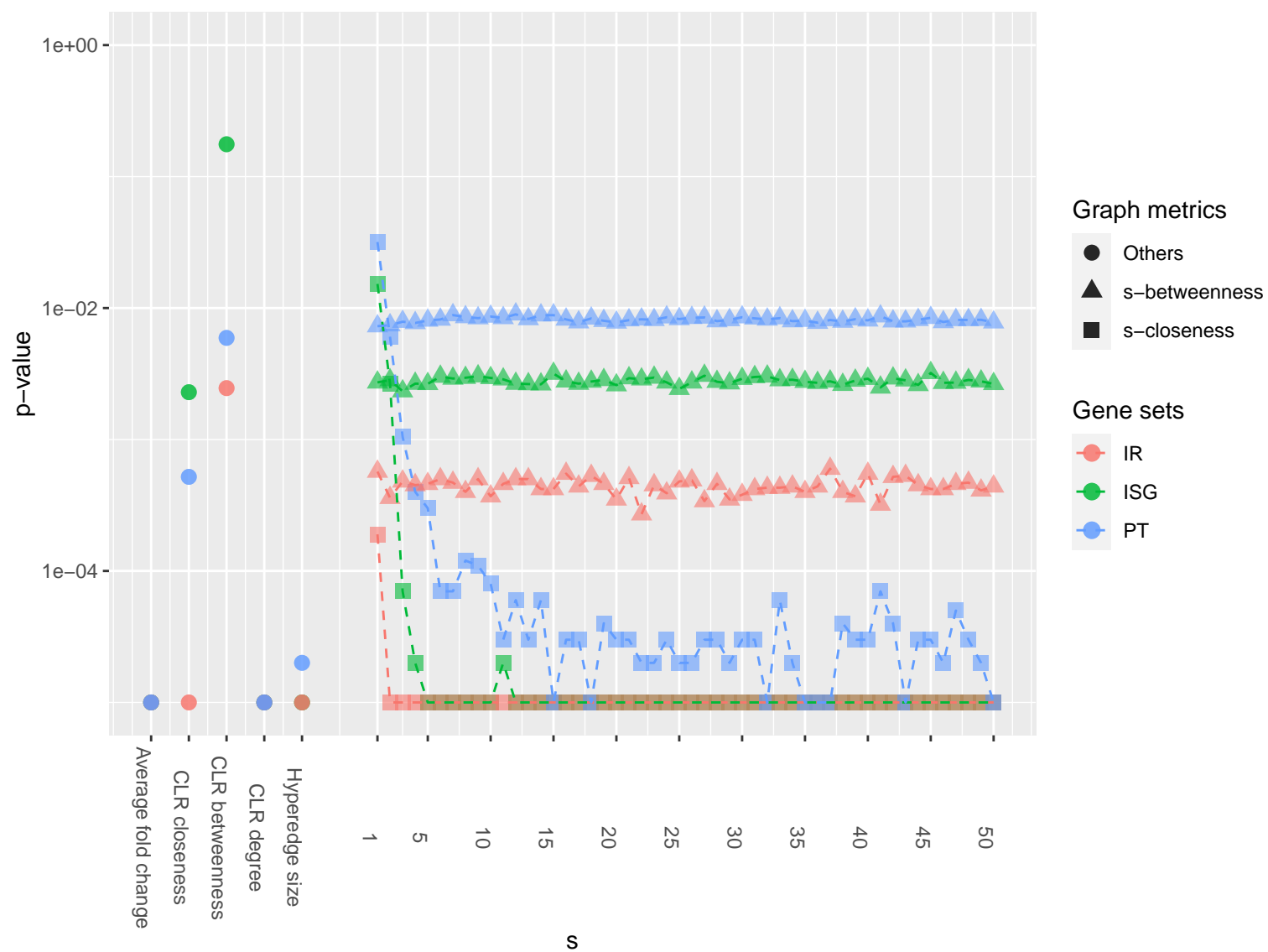

Supplement: Supplementary file 3 — Additional file 3. These figure is analogous to Figure 4 for additional z-score threshold z. [file 12859_2021_4197_MOESM3_ESM.pdf]

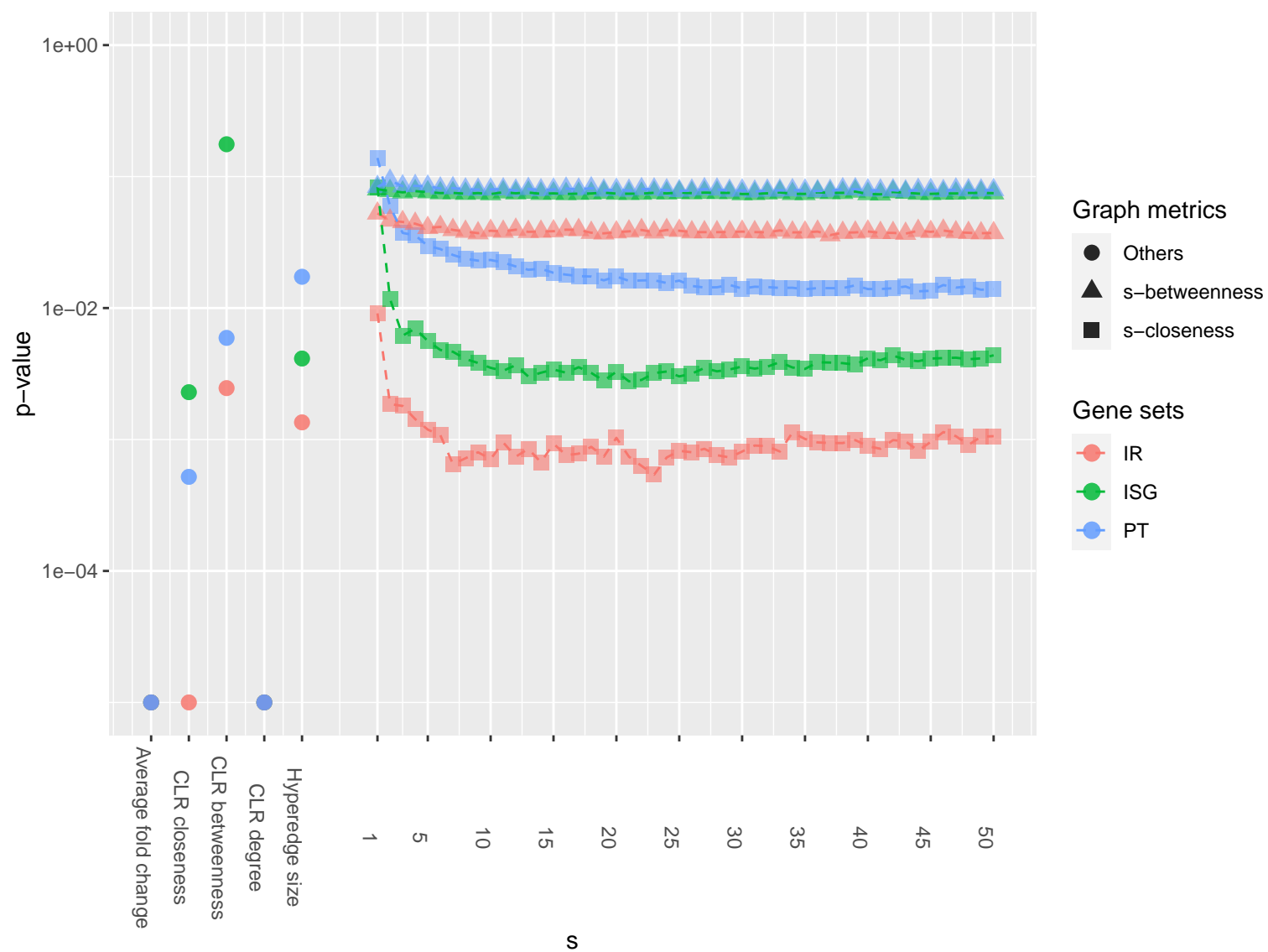

Supplement: Supplementary file 4 — Additional file 4. This figure is analogous to Figure 4 for additional z-score threshold z. [file 12859_2021_4197_MOESM4_ESM.pdf]

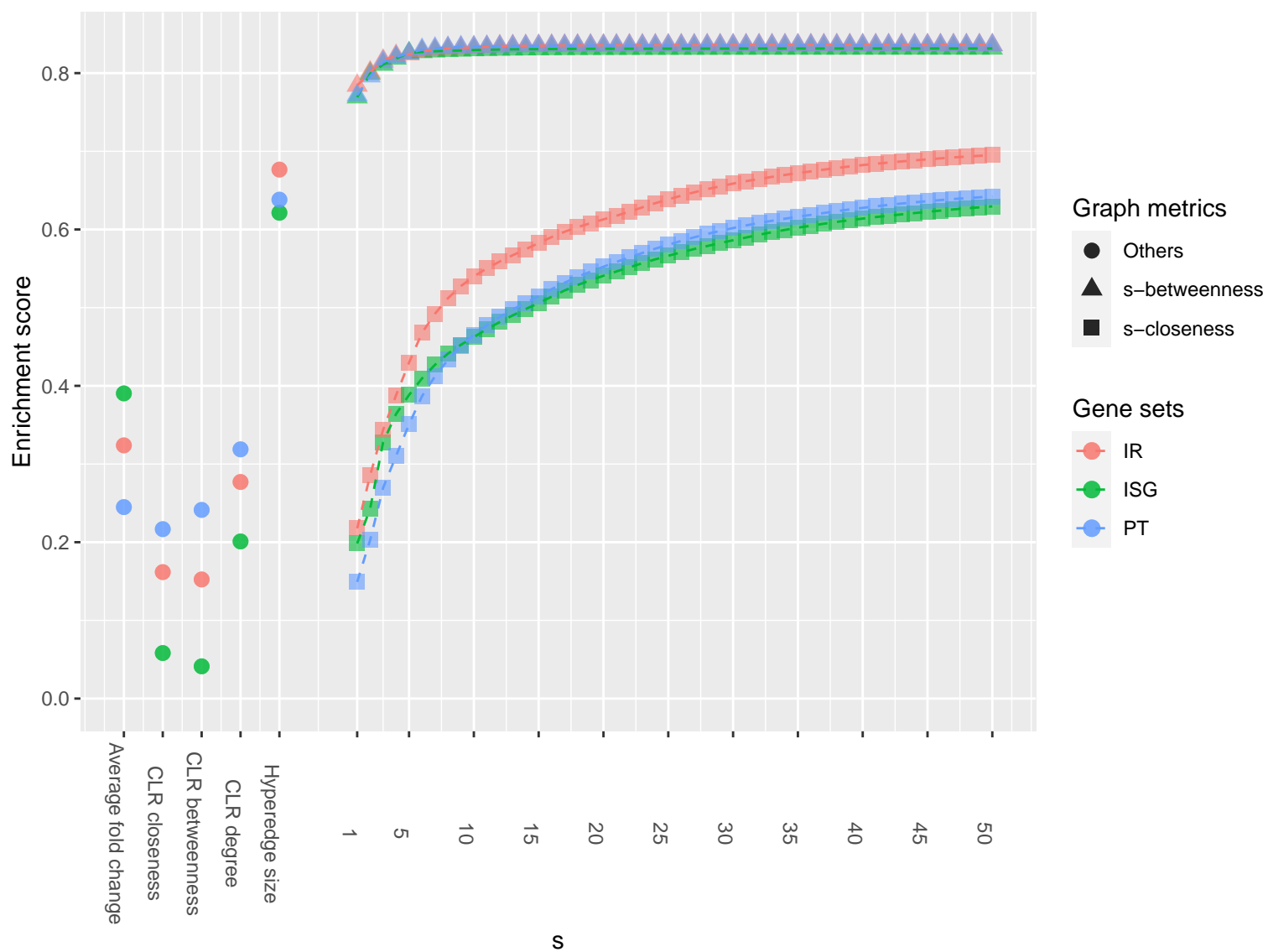

Supplement: Supplementary file 5 — Additional file 5. This figure shows the p-values for the GSEA enrichment scores (shown in Figure 4) for z-score theshold z. [file 12859_2021_4197_MOESM5_ESM.pdf]

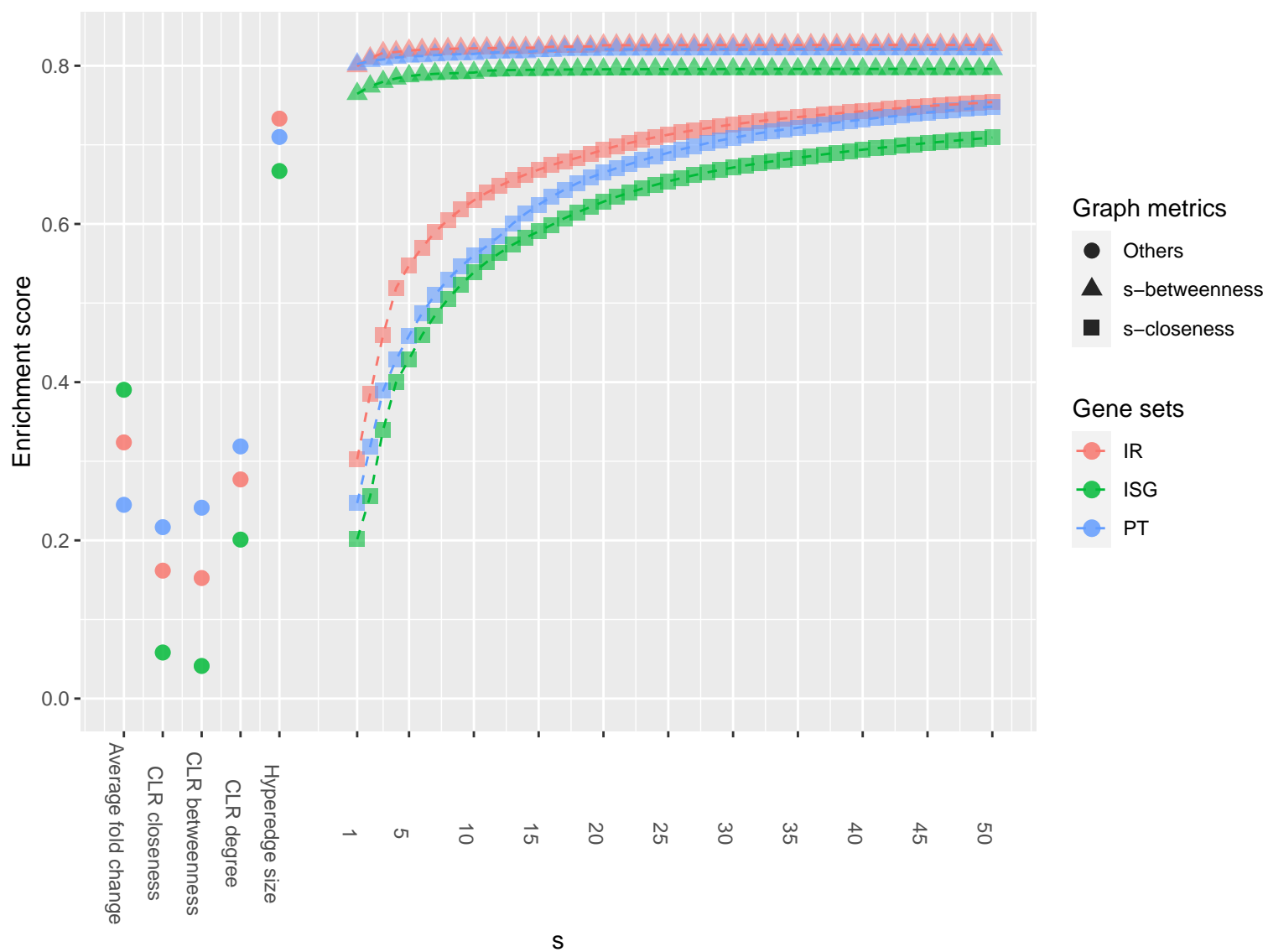

Supplement: Supplementary file 6 — Additional file 6. This figure shows the p-values for the GSEA enrichment scores (shown in Figure S1) for z-score theshold z. [file 12859_2021_4197_MOESM6_ESM.pdf]

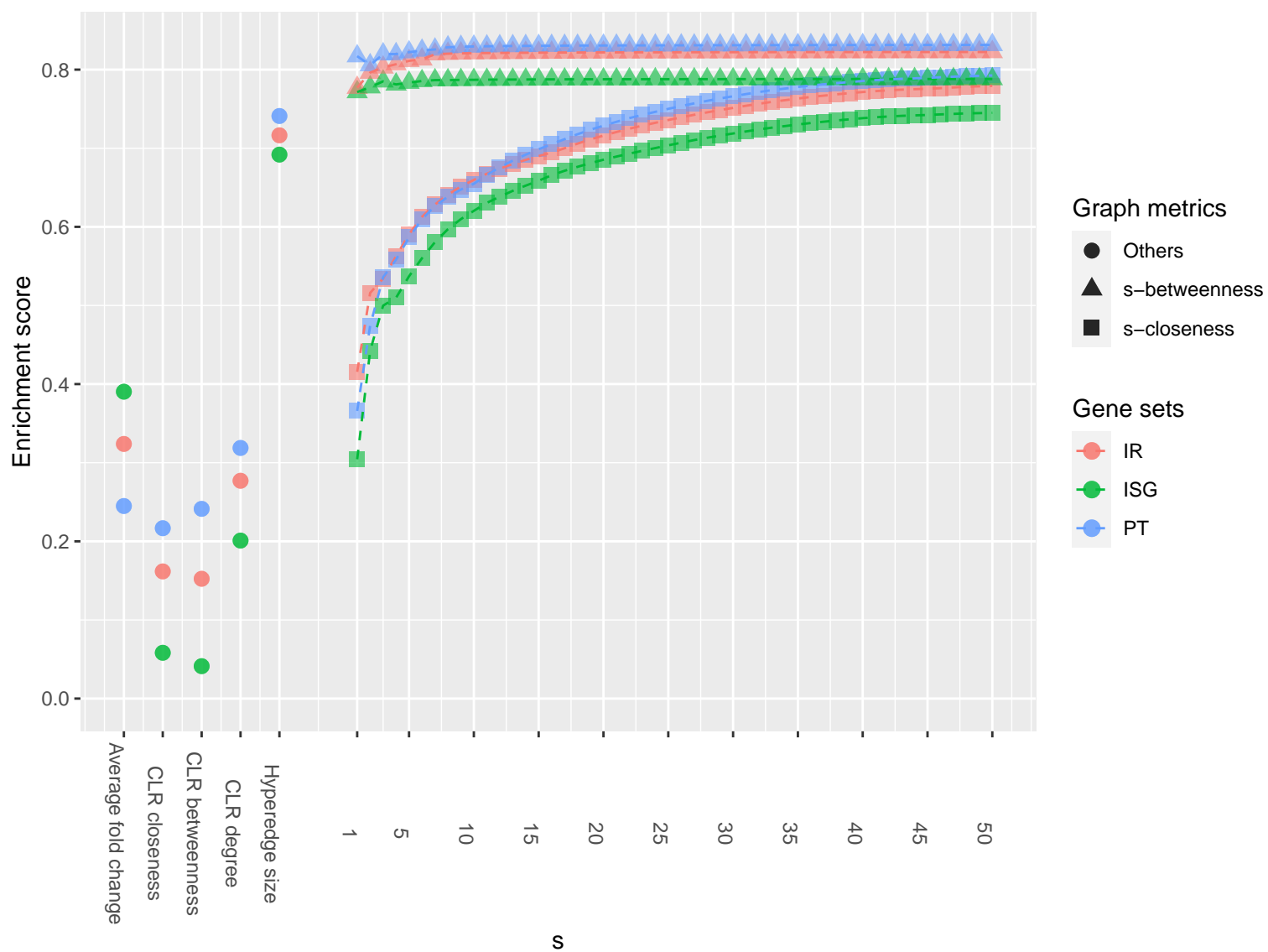

Supplement: Supplementary file 7 — Additional file 7. This figure shows the p-values for the GSEA enrichment scores (shown in Figure S2) for z-score theshold z. [file 12859_2021_4197_MOESM7_ESM.pdf]

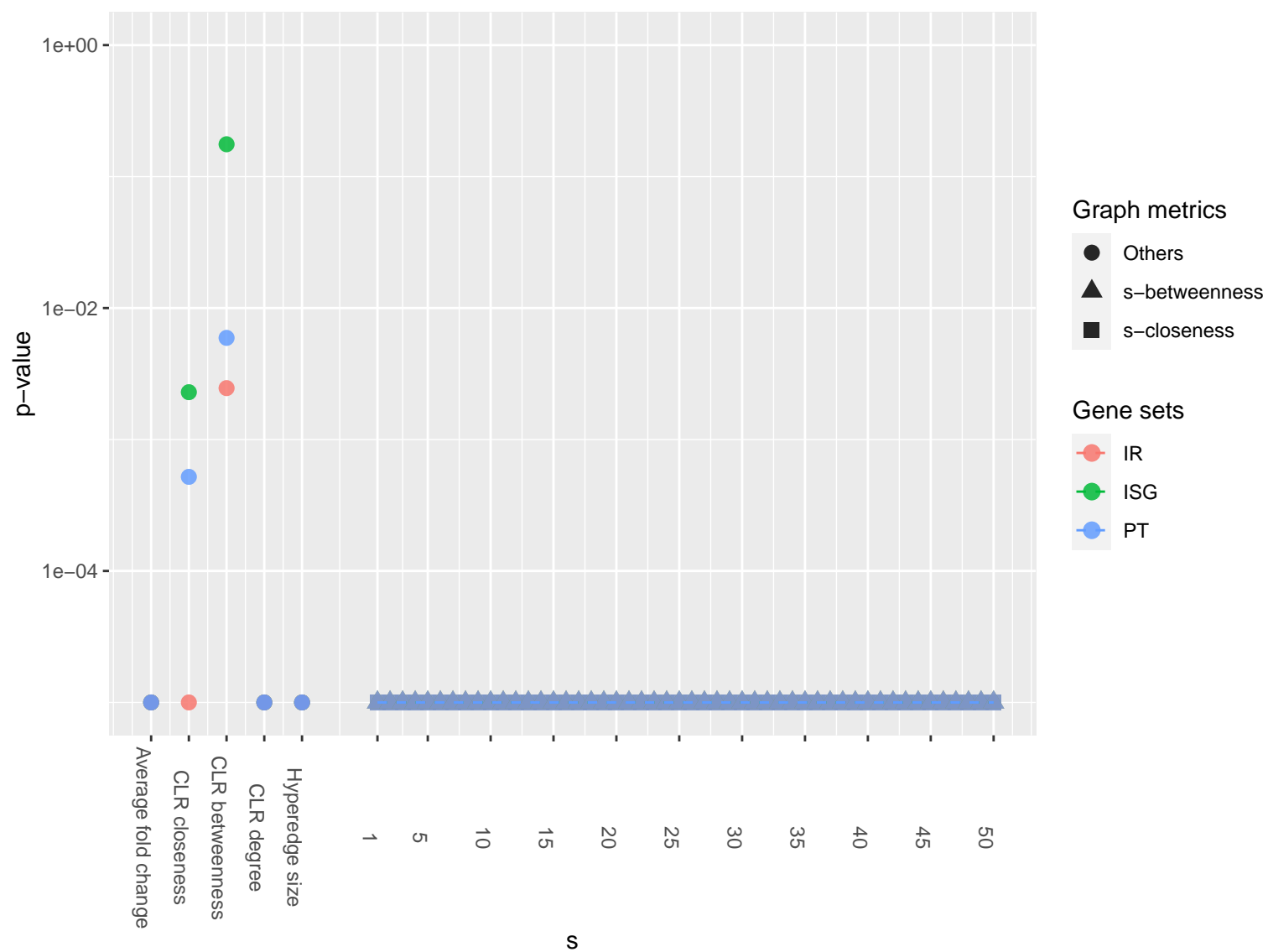

Supplement: Supplementary file 8 — Additional file 8. This figure shows the p-values for the GSEA enrichment scores (shown in Figure S3) for z-score theshold z. [file 12859_2021_4197_MOESM8_ESM.pdf]
